# Career Adaptability and Resilience of Mental Health Service Users: The Role of Career Counseling

**DOI:** 10.3390/bs13110886

**Published:** 2023-10-26

**Authors:** Nikos Drosos, Antonis Korfiatis

**Affiliations:** 1 Department of Social and Behavior Sciences, School of Humanities, Social and Education Sciences, European University Cyprus, Nicosia 2404, Cyprus; 2 PanHellenic Association for Psychosocial Rehabilitation and Work Integration (PEPSAEE), 10439 Athens, Greece; a.korfiatis@pepsaee.gr

**Keywords:** career adaptability, resilience, mental health service users, IPS model

## Abstract

The employment rate of people who face severe mental health issues is extremely low, while the vast majority expresses their willingness to work. There are various obstacles that impede their work re-integration process. Apart from the illnesses’ symptoms and the employers’ stigma, these barriers are strongly associated with the effects of long-term unemployment and the lack of positive psychosocial resources, such as career adaptability and resilience. The present study aims to investigate career adaptability and the resilience of mental health service users who receive career counseling services. The career counseling approach that was used combines elements from the IPS model and the career construction approach that has been developed to address the contemporary world of work challenges. We investigated how mental health service users view themselves in terms of career adaptability and resilience, and which factors contributed to their development or impeded them. We used a qualitative approach, which allows for an in-depth exploration of the participants’ views. Fifteen mental health users who receive career counseling services were interviewed. The results showed that mental health service users believe that they can overcome any difficulties and setbacks when they have adequate support from their social network and when they receive career counseling services. They highlighted the importance of counseling services to maintain their work and cope with stressful events. Further implications of the results regarding vocational rehabilitation of mental health users as means for social inclusion are discussed.

## 1. Introduction

The present study aims to investigate the positive psychosocial resources that people with mental health disorders use when facing work challenges and transitions. By “mental health disorders”, we refer to severe mental health issues which have a significant effect on people’s lives by causing impairment in important sectors of their functioning. These kind of severe disorders include psychotic disorders, such as schizophrenia, or some forms of mood disorders, such as bipolar disorder. When people receive treatment, the symptoms of the disorders stop or decrease significantly and they are quite capable of being productive and working effectively [1]. Nonetheless, despite their ability to work and the fact that work is a critical aspect of social inclusion, the employment rate of people with severe mental health issues is less than 30% [2], which is by far the lowest among people with disabilities. From this point onwards, the term ‘mental health service users’ will be used in the paper as it highlights the conscious choice of the individual to seek and receive mental health services rather than the terms ‘person with mental health problems/mental disorder’ or ‘mentally ill’ which have negative connotations.

Several studies have demonstrated that mental health service users have the will and desire to work [3]. This desire does not simply stem from the potential improvement of their financial situation, but reflects the broader impact of work on individuals’ lives [4]. Having a job implies a structured daily life, increased life satisfaction, increased sense of self-worth, increased social contact, and the sense of being a productive member of society. Furthermore, being employed is an essential step towards one’s recovery as it has multiple clinical benefits such as reducing both symptoms of the illness and relapses [5]. Therefore, we have thousands of published recovery stories from users that highlight the importance of being employed in overcoming illness and achieving a meaningful life, e.g., [6]. However, mental health service users’ work integration is significantly hampered by various barriers: stereotypes of employers and society [7,8], low expectations of mental health professionals [9], and internal barriers and lack of skills due to long-term unemployment and/or society’s stereotypes. Mental health problems are closely associated with long-term unemployment and its negative consequences. People tend to have low self-esteem and self-efficacy beliefs, as well as a greater fear of failure [10] which often prevents them from trying new situations. Low career adaptability and resilience contributes to a vicious cycle, leading to work exclusion. 

### 1.1. Career Adaptability and Resilience

The modern labor market is characterized by transitions and changes. Both career adaptability and career resilience are concepts developed to highlight the different ways in which individuals react when faced with such transitions, changes, and/or difficulties [11,12]. Some people are unable to cope with change, while others can and do continue to function and work productively even in stressful situations. Also, some people are better prepared than others so that transitions and changes come more smoothly.

The concept of career adaptability refers to an individual’s readiness to cope with changes and difficulties in his/her work life. According to Savickas [13,14], it has the following dimensions: (a) concern (interest/concern about one’s career future), (b) control (sense of control over that future based on one’s own actions), (c) curiosity (exploratory behavior to gather information), and (d) confidence (one’s belief that difficulties can be overcome). On the other hand, career resilience refers to the individual’s quick recovery despite adverse situations and effective management of changes, difficulties, and stressful events. It can be described as one’s willingness and ability to successfully cope with adverse career situations and recover after changes and career shocks [15]. 

Both concepts (career adaptability and career resilience) came about in response to the fact that individuals face transitions, challenges, and stressful situations and events in their careers. Adaptability refers more to an individual’s readiness to cope with such situations, i.e., effective prevention, and resilience refers to the individual’s good functioning despite the stressful and challenging situations, i.e., effective response [16]. Nevertheless, career adaptability is positively related to adaptive behaviors and beliefs, which the individual assumes when facing career transitions, changes, or other tasks [17,18]. These behaviors and beliefs (which show career resilience) mediate between career adaptability and life satisfaction. Therefore, there is a strong positive correlation between the two concepts and it appears that adaptability leads to resilience [19]. 

### 1.2. The PEPSAEE Career Intervention Model for Mental Health Service Users

Admittedly, at an international level, the most successful model that has been used for the work integration of people with mental health problems is the Individual Placement and Support (IPS) model [4,20,21,22,23]. This model can be categorized as part of the broader ‘supported employment’ interventions. The main features of supported employment models are: (a) time-unlimited support for both job seekers and employers (and therefore requires networking and collaboration with employers), (b) emphasis on paid employment with a satisfactory wage for the specific job, and (c) the integration of the individual into work in the open market on the same terms and conditions as other workers [24]. The individuals are also supported in order to maintain their job after being hired. In particular, the IPS model places emphasis on close cooperation between career practitioners and other mental health specialists in order to offer quality and coherent services, and on the rapid (but not hasty) integration of the individual into jobs that meet his/her own preferences and occupational profile.

In Greece, the first substantial effort to implement supported employment was the establishment of a “Support for Employment” office by PEPSAEE, a Scientific Not-for-Profit Organization, in 2010 and, subsequently, the implementation of the “Bridges for Employment” project (2012–2014), which provided the pilot operation of 15 supported employment offices in mental health units [25]. In 2023, the Greek National Plan for Mental Health was published by the Ministry of Health and it foresees the creation of seven more supported employment offices, but this remains to be implemented. The “Support for Employment” office of PEPSAEE provides services to more than 300 mental health service users annually aiming at work integration/reintegration and job retention [25].

PEPSAEE developed and implemented an intervention model that combines the IPS model with contemporary career theories based on constructivism. More specifically, it makes use of the life design paradigm [26], which was developed to address the challenges of the contemporary world of work and derives from the self-construction [27] and career construction [13] theories. The LD paradigm considers the person as a “whole” with work context being just one of its components, and therefore addresses all aspects of the person’s life, aiming at supporting him/her to achieve a satisfactory career and life. Career adaptability is considered one of the most important resources in the life designing process and, therefore, career interventions based on the life design paradigm aim to foster it [28]. This can be achieved both via individual career counseling and via group career interventions [29]. As subjective career stories can foster or impede career adaptability, in individual counseling, people are assisted in constructing career stories that recognize the challenges that they have faced and overcome in the past recovering from adversity. Stories that show pessimism, defeatism, and helplessness are deconstructed and substituted with stories that steer adaptive attitudes and behaviors and promote an agentic role for the person in his/her career construction. Moreover, career counselors can use various activities to promote the four elements of career adaptability: orientation activities for career concern, decision-making activities for career control, information-gathering activities for curiosity, and vicarious learning/modeling for career confidence [29]. Furthermore, group interventions based on the life design paradigm have been developed and show very good results [30].

The PEPSAEE career intervention model recognizes that although acquiring a job that matches the mental health service user’s career interests and skills and has a reasonable salary are two very important aspects, they are not enough. Without adequate focus in career planning, mental health service users may move “laterally” in the labor market without advancing their careers. Prioritizing just job placement without emphasis on long-term career plans and the development of adaptability and resilience might result in short job tenures or in low-wage jobs [31]. Therefore, individuals are encouraged to explore the meaning that they attribute to work and career and to construct or re-construct their career stories. As previously stated, career stories that highlight successful recoveries from previous adversities foster career adaptability and resilience. The PEPSAEE model [25] encompasses the main characteristics of IPS, while giving emphasis to developing short- and long-term career goals and in fostering career adaptability and resilience. Career counseling services focus on: (a) motivating the individual, (b) attributing new meanings in the individual’s current work history and constructing a new positive (subjective) career story, (c) developing self-efficacy, career adaptability, and resilience, (d) reconstructing dysfunctional thoughts and beliefs about their career, (e) training in job search techniques, developing self-presentation skills, and (f) enhancing their vocational self-awareness so that they can choose the job or education that is right for them. With the help of the counsellor, the user formulates an action plan to achieve his/her work goals and the counsellor supports him/her in its implementation without any time limit. Support is provided to both the individual and the employer in order for the first to maintain his/her job and overcome any difficulties or challenges. Moreover, group interventions take place aiming at fostering career adaptability, career resilience, and other resources and skills. Furthermore, there is a strong emphasis on cooperation with other mental health services and networking with employers, aiming to create a friendlier environment for the recruitment of mental health service users [25].

### 1.3. The Present Study

Although it has been well established that both individual and group career counselling services can foster career adaptability and resilience of individuals, to our knowledge no such study has been conducted regarding mental health service users.

The aim of this study is to explore the career adaptability and career resilience of people with severe psychiatric disorders who are employed and, at the same time, receive career counselling services. More specifically, the present study will explore: (a) how individuals cope with the emotions that they feel when faced with changes or stressful and difficult situations and events at work, (b) how they cope with these changes and/or difficult situations, (c) whether they feel that through their own actions they can successfully manage these events and whether they believe that any difficulties can be overcome, (d) the factors that facilitate and the factors that hinder the successful management of these events, and (e) the role of career counselling services in coping with difficulties and setbacks.

## 2. Materials and Methods

### 2.1. Research Design

For the design, operation, and analysis of this study, the qualitative methodology was used, which was considered the most suitable to address both the research questions and the characteristics of the mental health service users.

The research design started with the formulation of the research questions. The general interest of the researchers was related to the responses of mental health service users when facing challenges or transitions related to their work. After a discussion among the researchers, the interest turned into the five research questions of the study.

### 2.2. Tools and Data Collection

The semi-structured interview method was used to collect data, which is the most widely used method of data collection in qualitative research. Keeping in mind that the interview questions should be linked to the main research questions of the study while allowing the participants to narrate their experience, the researchers created an interview guide which includes 10 questions. To form the questions, we made use of the items of the Career Adaptabilities Scale [17], which is widely used to assess career adaptability. We reviewed the items with the assistance of two mental health service users to make sure that the phrasing of the interview questions was appropriate. Nonetheless, asking the users their attitudes and potential behaviors towards possible transitions, changes, and stressful events seemed to be confusing as the two users reported that it was difficult to visualize them in real-life situations. Therefore, we opted to ask the participants to describe the last time that they faced a disrupting change, transition, or event; we asked more questions regarding their attitudes, thoughts, emotions, and coping behaviors towards this particular event (bearing in mind the components of the aforementioned scale that we wanted to measure).

All questions were created to gather the necessary information in an open, non-directive way. Finally, some demographic questions and a consent form to participate in the survey were added to the guide. The interviews were conducted face-to-face and they were recorded. Fifteen recordings were collected.

### 2.3. Participants

There were 15 participants of the survey: 8 males and 7 females. All participants are mental health service users, reside in the community, are employed, and receive career counseling services to maintain the job they have. Participants have been diagnosed with schizophrenia and delusional disorders. They were approached to participate in the research face-to-face, where the researchers described the study and its aims and obtained their consent. 

### 2.4. Research Data

For data analysis, the method of thematic analysis was utilized, which according to Braun & Clarke [32] is a method of identifying, analyzing, and transferring patterns of meaning within qualitative data. In the process of analysis, we followed the six steps that Braun & Clarke [32] suggest: 1. familiarizing yourself with the data, 2. generating initial codes, 3. scanning for themes, 4. re-examining themes, 5. clarifying and naming themes, and 6. creating the report. Researchers familiarized themselves with the data by individually studying the recordings/transcripts and doing an initial coding bearing in mind the research questions. Subsequently, they discussed with each other and exchanged experiences, detecting common themes. Then, they reviewed the themes individually and came back collectively, clarifying and labeling the themes. Finally, a report of the results was created, discussed among the researchers, and finalized in its final form. The final form presents the themes that emerged by linking them to the research questions. 

The conclusions of the study were reached after discussion among the researchers considering the research questions, the results of the study, and the literature review.

## 3. Results

### 3.1. Projecting Oneself into the Future (Career Concern)

We explored the extent to which participants envision their career future and set long-term career goals.

A prevalent theme was *lack of future projection*. Almost half of them said that they do not think about the distant future, possibly reflecting their own past disappointments (due to relapses) in plans they had made for the future:

“I don’t know what is waiting for me, here I don’t know what is waiting for me tomorrow, I can’t think about the future, I don’t want to make changes, I don’t think about the future”

(P., male, 47 years old)

Another theme was *lack of career advancement* when envisioning the future. Some people set having a job as their main goal for the future without concern regarding their development in it or experiencing satisfaction through their work. This may also be related to their own past frustrations due to the illness:

“[…] to try to survive, because from a young age I was always being chased out of jobs […] to survive in any jobs”

(H., female, 49 years old)

Moreover, another theme was *concern for mental health*. Several participants emphasized the importance of their personal development in the future, but focused only on mental health issues:

“I am starting to think positively… I have to change as a person if we want to leave the past behind… I will change as a person… it is good to evolve for the better”

(F., female, 42 years old)

Finally, the last theme that was introduced by a few participants was *finding a suitable career*. They referred to their career development and finding a job that would be more satisfying for them:

“[…] I have thought to change something… I’m thinking of leaving the nursery and going to work in a garbage truck because the nursery has too much responsibility… I don’t like it”

(N., male, 33 years old)

### 3.2. Considering the Future Controllable (Career Control)

Furthermore, we examined the issues that emerged regarding mental health service users’ beliefs for the control they have over their future. 

Approximately half of the participants initially stated that in case unexpected difficulties or challenges arise they cannot overcome them:

“[…] I don’t feel that by my own actions I can solve the problems”

(H., female, 49 years old)

However, it subsequently appeared that they rather wanted to emphasize the need for sources of support and for gradual familiarization with the new challenges rather than actual belief that they cannot overcome the challenges and setbacks. Therefore, two main themes that emerged were *sources of support* and *gradual familiarization with the setbacks*:

“When there are changes at the beginning I feel that I cannot cope with them but then with a lot of effort and slowly I overcome whatever difficulty there is”

(K., female, 47 years old)

Many stressed that they believe that every difficulty can be overcome and mentioned the following as the main *sources of support*: (a) their social network, (b) their previous experiences, and (c) activation and taking action:

“Not on my own… I needed to talk to my best friend”

(H., female, 64 years old)

“The truth is that the experience I have gained helps me to cope with difficulties…… because I now have the experience”

(P., male, 47 years old)

“I will fight with my own knowledge and my own effort to solve the problem […] I will persevere and I will fight to solve the problem”

(F. female, 42 years old)

### 3.3. Propensity to Explore the Environment (Career Curiosity)

As expected, most participants reported negative emotions when facing changes, challenges, or unexpected events (fear, increased negative thoughts, feeling of loss of control, stress, and anxiety), which also lead to psychosomatic symptoms:

“I get anxious and think about the changes all the time. I am constantly thinking and feel anxiety. The changes, if they have serious consequences, can even leave me sleepless [….]”

(G., male, 45 years old)

“I feel very bad, I feel that I won’t be able to cope, I get very stressed, I get stressed, sometimes I get psychosomatic like diarrhea”

(P., male, 47 years old)

Nonetheless, there is a gradual reduction in the intensity of negative emotions through becoming familiar with the change or situation. Therefore, a main theme that emerged was seeking information as a way to manage the negative emotions and subsequently to explore possible solutions:

“[…] At first I feel strange because I am a person with anxiety… whenever I get some anxiety… but then when I start… e.g., a new project is announced, when I read the invitation, I discuss it with colleagues and organise it gradually… This anxiety is therefore reduced… the process goes more smoothly.”

(A. female, 51 years old)

A second theme that emerged was *seeking support and help*. This help was asked for (and, subsequently, provided) both from the careers counsellors and from colleagues at work. Therefore, when facing a setback or challenge, participants use their social network as a valuable source of information and as a way to get guidance on how to seek the necessary information:

“Only by asking for help and taking time to adjust… Only by asking the people in charge can you move forward because when you are alone you can make a wrong or hasty move… the other person unblocks you, they are the third eye”

(L. female, 57 years old)

### 3.4. Belief in One’s Own Ability to Overcome Challenges (Career Confidence) and Successful Ways of Coping (Career Resilience)

In contrast to the negative feelings and situations, some participants talked about the belief that difficulties will be overcome and there will be a positive outcome based on their previous experiences on overcoming obstacles, which shows their *career confidence*. As mentioned before, one’s career story and the meaning he/she attributes to this story (emphasis on the learning gained from previous experiences versus defeatism) have a very important role on the way he/she believes that they can cope with challenges. Therefore, a theme that emerged was the *meaning attributed to past experiences*.

“It’s true that when I heard it I had a hard time… but I’ve been through so much… I’m sure I’ll be able to figure it out. […]”

(D. female, 53 years old)

Moreover, we explored the mechanisms that individuals use to cope with changes and difficult situations. Although there were separate questions on coping with changes and coping with difficult situations, the participants’ responses were the same.

Two different areas of response and management seemed to emerge from the individuals’ responses: (a) *managing the emotions caused by the change* and (b) *managing the event itself* so that the individual can continue to function satisfactorily.

In relation to managing both the negative emotions and the stressful event, a key role is played by *social support* from the individual’s network of informal caregivers (family, friends, and so on) and from the career counsellor. Almost all participants stressed its importance. It seems that when individuals experience difficulties, social support is the most important factor in their successful management, while lack of it is the biggest barrier:

“…You talk to your own people…you don’t leave it to the mercy of God… You talk and you find a solution […]”

(E. female, 36 years old)

“…I talk to the career counsellor…. He reassures me and I see things more logically […]”

(D. female, 53 years old)

“ […] others help a lot… my son helps me… I need this support”

(H., female, 64 years old)

In relation to managing the change itself, a theme that emerged was the *individual’s personal effort* to find appropriate solutions (which is linked to the gradual reduction in negative feelings and the belief that any difficulties can be overcome). Several participants reported that after a while they started to think about ways of coping with difficulties.

“I struggle and I try to manage them properly… I try to deal with them in an organised way; I try to make the necessary changes… I try to achieve my goal more easily, not to be taken down…”

(P. male, 47 years old)

Finally, several people reported that the most important thing when dealing with challenges and setbacks is to “not lose hope” and so they try to think more positively in order to visualize possible solutions. Therefore, *positive thinking* emerged as a factor in successfully coping with change; although, possibly, this is a result of both the career counselling and other mental health services they receive.

“I try to change my way of thinking, to see everything in a positive way and say that I will try harder”

(K. female, 47 years old)

“It helps me to look at some things positively in terms of my health. I try to use a positive outlook and attitude to smooth things over”

(R., male, 46 years old)

### 3.5. The Role of Career Counselling Services in Successful Coping

The majority of participants stressed the importance of continuous support from the counsellor as they feel that whatever difficulties they encounter, they should not face them alone. A main theme that emerged was *sharing the burden:*

“It helps me to understand that I am not alone, that I am not the only one with problems, others have problems too and that calms me down”

(H., female, 49 years old)

Furthermore, career counselling helps the person to *acquire different perspectives* regarding the challenges that he/she faces and offers guidance even on practical issues:

“It is very important for me that when I need support I pick up a phone and discuss it with the counsellor. My career counsellor will show me another perspective”

(D., male, 40 years old)

Several people highlighted the following as important elements for their support: (a) no time limit on support and regular collaboration with the counsellor, (b) group counselling, (c) support from other mental health professionals, and (d) support and encouragement from people in their immediate environment. The above shows both the necessity of supporting the individual to maintain work without time limitations, as envisaged by the IPS model, but also the importance of group interventions and sharing of experiences between users, of working with other mental health professionals to provide holistic and coherent services, and the necessity of providing support and guidance to the informal caregivers of the individuals to help them in their work integration as well.

“I would like to do group therapy […] it will help me to become a better person”

(J., female, 57 years old)

“What I would like is … whenever I am not well … to have an easy and direct intervention from mental health professionals”

(P., male, 47 years old)

“To be supported by your own people […] I need it”

(L., female, 36 years old)

## 4. Discussion

The purpose of this study was twofold. On the one hand, we wanted to explore how mental health service users working in the open labour market cope with the various challenges, changes, and barriers they encounter at work and whether they exhibit attitudes and behaviors that show career adaptability and resilience. On the other hand, we wanted to look at the factors that help them to successfully cope with difficulties and in particular the role of the support they receive from their careers counsellor. The results of the study show that mental health service users exhibit readiness to address the various transitions, challenges, and setbacks that occur in the workplace, i.e., they have career adaptability and they manage to effectively cope, and they exhibit career resilience.

The component of career adaptability which they mostly seem to lack is career concern. Career concern refers to visualization of their career future and setting long-term career goals. Our study has shown that many mental health service users avoid making long-term plans for their work future and career advancement. This may be due to past disappointments and frustrations that have been caused by relapses of their illness. Although this behavior protects them from potential new disappointments, their lack of planning keeps them from moving up the career ladder and achieving a satisfying career. This finding is in line with previous studies [33,34] with people with disabilities, which showed a lack of concern regarding their career future and long-term goals. Therefore, it is important for career counsellors to assist the individual in envisioning their vocational future, bearing in mind that there is always the possibility that unexpected events arise and upset the planning. The life design paradigm provides the necessary framework and practical tools to deconstruct and reconstruct the individual’s personal career story and help him/her more broadly re-imagine his/her career path [26,29]. Our study showed that some participants derived confidence in overcoming obstacles from the fact that they have managed to overcome major difficulties in the past. Therefore, career counselors should make more use of activities designed to help mental health service users in attributing new (positive) senses and meanings in past experiences [35] and subsequently envision the future they want for themselves.

Our study also showed that mental health service users feel that they have some level of control of their career and they exhibit behaviors that show career curiosity, the second and third components of career adaptability, when they face career challenges. Changes and obstacles at work usually cause negative feelings of stress and anxiety and people may feel helpless, but these feelings are temporary and only last a short time. As time passes by and individuals become more familiar with the stressful event, they seek more information to overcome the obstacles. Their social network (employers, career counselors, and informal caregivers) plays a crucial role in helping them gather the necessary information and find appropriate ways to cope with the event. In the next paragraphs, we will further examine the implications of the importance of the social network for the career counseling services. We should note that the career counseling services that participants received included many information-seeking activities aimed exactly at fostering career curiosity.

Furthermore, the results of the study showed the importance of positive thinking and optimism. Several participants referred to their own personal efforts to remain optimistic in the face of adversity and to see situations from different perspectives. This way of thinking is often the result of both individuals’ life experiences and successful management of difficulties in the past and the support they have received from career counsellors and other mental health professionals. As mentioned previously, the life design paradigm offers the framework and tools to assist people in attributing new meanings in their past experiences and therefore it fosters optimism and adaptability [36]. Moreover, our study highlights the importance of collaboration between career counsellors and other mental health professionals to provide more holistic and coherent services to individuals. In this way, work issues are placed within the broader context of the individual’s life and meaningful recovery leading to true social inclusion. This finding is in line with the work of Drake et al. [37], who argue that a team-based approach is particularly helpful to people with serious mental disorders as they face a multitude of different issues.

According to our results, mental health service users feel rather confident that they will be able to overcome challenges and obstacles (the fourth component of career adaptability), and after the first shock, they begin to look for ways to manage it successfully. As previously mentioned, in order to successfully cope with these difficulties, all participants placed particular emphasis on the support they receive (and need) from their social network. It appears that this support was essential both for successfully managing the negative emotions caused by the change and for successfully coping with the stressful event itself afterwards. As the research showed, the support derives from three separate groups: (a) support from the close social network (informal caregivers, family members, and friends), (b) from employers and colleagues at work, and (c) from the career counsellor. Therefore, we argue that career counseling services should work with all three groups: mental health service users themselves, employers and other employees, and informal caregivers.

The role of informal caregivers is particularly important as these are the people who are with mental health service users on a daily basis. They often face difficulties and frustrations themselves [38], are not aware of the specifics of mental illness, and do not know how to offer better support to individuals to enable them to overcome any work barriers they face. It would therefore be good in the context of career counseling services to place more emphasis on supporting informal caregivers. This finding is in line with other previous works, e.g., [35]. This support could take the form of the implementation of empowerment groups for informal caregivers, but also psychoeducation groups focusing on the vocational rehabilitation of individuals. 

Furthermore, the importance of preparing the individual’s workplace and colleagues to be supportive when the mental health service user is experiencing a difficulty was also evident. According to the IPS model of supported employment, counselors should offer support to employers and facilitate the individual’s entrance in the workplace [4]. The results of our study indicate that even more emphasis should be placed in networking with employers to create a friendlier environment for mental health service users. This preparation can take the form of workplace empowerment groups and brief training sessions on mental health issues. In this way, the work context will become more supportive while avoiding the risk of becoming overprotective and perpetuating the negative stereotype that mental health service users are not productive and effective in their work.

Finally, the great importance of regular and time-unlimited follow-up sessions between the individual and the career counsellor was highlighted by the results of our study. According to the IPS model, support is provided to the individuals (and their employers) with no time limit to support them to maintain their job [4]. Work integration is an ongoing effort and when the individual is facing stressful situations, it is particularly helpful to be able to share concerns and feelings with his/her counselor. The career counsellor can help him/her manage his/her feelings and see the issues from other perspectives that will help him/her find solutions. In this way, successfully coping with challenges and setbacks is achieved and the person stays at work for a long time. 

### Limitations

Our study has some limitations. First, all participants were employed for more than twelve months at the time the interviews took place and, therefore, one could argue that they might have more career adaptability and resilience than their counterparts that had not managed to maintain their job for so long. Nevertheless, the vast majority of people who have managed to get into work via the “Support for Employment” office stay employed for over 12 months and often for several years. Secondly, the study took place in a specific socioeconomic environment of one country (Greece), and therefore, results should be viewed with caution. Third, all data were obtained via self-report methods from the mental health service users, and we had no data from other sources such as their employers, colleagues, informal caregivers, or counselors. Future studies could explore the perspectives of their social network.

## Data Availability

Data are unavailable due to privacy. Nonetheless, authors can be contacted and provide data to researchers that are interested.
